# Tamoxifen induces pleiotrophic changes in mammary stroma resulting in extracellular matrix that suppresses transformed phenotypes

**DOI:** 10.1186/bcr2220

**Published:** 2009-01-27

**Authors:** Rhonda Hattar, Ori Maller, Shauntae McDaniel, Kirk C Hansen, Karla J Hedman, Traci R Lyons, Scott Lucia, R Storey Wilson, Pepper Schedin

**Affiliations:** 1 Department of Medicine, Division of Medical Oncology, University of Colorado Denver, 12801 East 17th Avenue, Aurora, CO 80045, USA; 2 Program in Cell Biology, Stem Cells and Development, University of Colorado Denver, 12801 East 17th Avenue, Aurora, CO 80045, USA; 3 Program in Cancer Biology, University of Colorado Denver, 12801 East 17th Avenue, Aurora, CO 80045, USA; 4 Department of Pediatrics, University of Colorado Denver, 12801 East 17th Avenue, Aurora, CO 80045, USA; 5 University of Colorado Comprehensive Cancer Center, University of Colorado Denver, 12801 East 17th Avenue, Aurora, CO 80045, USA; 6 Department of Pathology, University of Colorado Denver, 12801 East 17th Avenue, Aurora, CO 80045, USA; 7 AMC Cancer Research Center, University of Colorado Denver, 12801 East 17th Avenue, Aurora, CO 80045, USA

## Abstract

**Introduction:**

The functional unit of the mammary gland has been defined as the epithelial cell plus its microenvironment, a hypothesis that predicts changes in epithelial cell function will be accompanied by concurrent changes in mammary stroma. To test this hypothesis, the question was addressed of whether mammary stroma is functionally altered by the anti-oestrogen drug tamoxifen.

**Methods:**

Forty female rats at 70 days of age were randomised to two groups of 20 and treated with 1.0 mg/kg tamoxifen or vehicle subcutaneously daily for 30 days, followed by a three-day wash out period. Mammary tissue was harvested and effects of tamoxifen on mammary epithelium and stroma determined.

**Results:**

As expected, tamoxifen suppressed mammary alveolar development and mammary epithelial cell proliferation. Primary mammary fibroblasts isolated from tamoxifen-treated rats displayed a three-fold decrease in motility and incorporated less fibronectin in their substratum in comparison to control fibroblasts; attributes indicative of fibroblast quiescence. Immunohistochemistry analysis of CD68, a macrophage lysosomal marker, demonstrated a reduction in macrophage infiltration in mammary glands of tamoxifen-treated rats. Proteomic analyses by mass spectrometry identified several extracellular matrix (ECM) proteins with expression levels with tamoxifen treatment that were validated by Western blot. Mammary tissue from tamoxifen-treated rats had decreased fibronectin and increased collagen 1 levels. Further, ECM proteolysis was reduced in tamoxifen-treated rats as detected by reductions in fibronectin, laminin 1, laminin 5 and collagen 1 cleavage fragments. Consistent with suppression in ECM proteolysis with tamoxifen treatment, matrix metalloproteinase-2 levels and activity were decreased. Biochemically extracted mammary ECM from tamoxifen-treated rats suppressed *in vitro *macrophage motility, which was rescued by the addition of proteolysed collagen or fibronectin. Mammary ECM from tamoxifen-treated rats also suppressed breast tumour cell motility, invasion and haptotaxis, reduced organoid size in 3-dimensional culture and blocked tumour promotion in an orthotopic xenograft model; effects which could be partially reversed by the addition of exogenous fibronectin.

**Conclusions:**

These data support the hypothesis that mammary stroma responds to tamoxifen treatment in concert with the epithelium and remodels to a microenvironment inhibitory to tumour cell progression. Reduced fibronectin levels and reduced ECM turnover appear to be hallmarks of the quiescent mammary microenvironment. These data may provide insight into attributes of a mammary microenvironment that facilitate tumour dormancy.

## Introduction

Once thought of as a passive support structure, the mammary microenvironment is composed of a complex mix of cellular, structural and soluble components capable of fundamentally altering mammary epithelial cell specificity and behaviour [1]. Consequently, the functional unit of the mammary gland is now recognised as the epithelial cell plus its extracellular matrix (ECM) and stromal and immune cells embedded therein [2]. Fibroblasts are primarily responsible for deposition of the stromal ECM. It is anticipated that for each organ fibroblasts deposit tissue-specific ECM [3]. The model of dynamic reciprocity postulates that the microenvironment, in particular the ECM, exerts an influence on gene expression in the mammary epithelial cell and, in turn, gene expression of the epithelial cell influences stromal cells and the composition of the ECM [2,4]. In support of this concept, our laboratory has shown that the composition of rat mammary ECM is dependent on reproductive state, demonstrating that the mammary microenvironment, as with the mammary epithelium, is under endocrine control [5]. Further, mammary ECM isolated from distinct hormonal states was found to facilitate epithelial cell proliferation, differentiation, death and glandular reorganisation in 3-dimensional (3D) cell culture, recapitulating events that occur *in vivo *with the pregnancy-involution cycle. Work by others has shown that the mammary ECM protein fibronectin and its specific integrin α5β1 are under hormonal control and in turn mediate hormone response in mammary epithelium, providing further support for the concept of Dynamic Reciprocity in the mammary gland [6,7].

Given the dynamic and reciprocal relation between ECM and normal mammary epithelial cells, it is not surprising that the microenvironment also exerts a significant effect on tumour cell behaviour [8]. Early evidence for stromal impact on cancer progression was observed by histological analyses; as wound healing-associated modifications in stroma, termed desmoplasia, were shown to contribute to poor prognosis in many human cancers, including breast, colon and prostate [9-14]. More surprisingly, even physiological changes in the mammary microenvironment have been demonstrated to influence tumour cell progression [5,15-17]. For example, mammary ECM isolated from mammary glands undergoing weaning-induced involution promotes breast tumour cell motility and invasion *in vitro *and metastasis in a xenograft model of breast cancer, whereas ECM isolated from quiescent virgin mammary tissue did not support these tumour cell attributes [15,17]. Mammary involution ECM is characterised in part by partial proteolysis of fibronectin and laminin, high-fibrillar collagen content, and increased matrix metalloproteinase (MMP) activity; all of which have been implicated in tumour progression [5,17,18]. Thus, evidence suggests that both pathological – and physiological-induced changes in mammary stroma contribute to breast cancer progression.

Whether the microenvironment can actively inhibit tumour progression has not been well studied. It is known that tumour cells can arrive at secondary sites in high numbers but fail to expand [19] and that microtumours and solitary tumour cells can reside in a dormant state for decades [20-22]. These data suggest that the microenvironment can indeed exert a significant protective effect. Experimental proof of this principle lies in studies in which malignant cells undergo a phenotypic reversion to polarised epithelium when exposed to a tissue-normalising ECM milieu [23-25]. Further, morphologically normal epithelial tissue adjacent to breast tumours can display loss of heterozygosity similar to that of the tumour, but without manifesting tumour cell characteristics [26]. One explanation is that malignant progression of these mutant, but morphologically normal, cells is inhibited by the local tissue microenvironment. Cumulatively, these data strongly suggest that the microenvironment can impart a dormant suppressive phenotype on malignant cells.

In this study, we tested the hypothesis that the stromal compartment responds with the epithelium to interventions that reduce breast cancer risk. Specifically, we addressed whether tamoxifen treatment alters the composition and function of mammary ECM in a manner consistent with supporting tumour cell dormancy. Tamoxifen is the endocrine treatment of choice for pre-menopausal women with breast cancer and a proven chemopreventive agent in high-risk patients [27]. Its canonical mechanism is as an anti-proliferative agent in oestrogen receptor (ER) positive mammary tumour cells [28]. Our data support the hypothesis that mammary stroma responds to tamoxifen treatment and remodels to an environment that would be inhibitory to breast cancer progression.

## Materials and methods

### Tamoxifen study experimental design

Forty female Sprague-Dawley rats, 73 days of age (Harlan-Teklad, Indianapolis, IN, USA) were randomised by weight into two groups of 20. Rats in the tamoxifen group were injected subcutaneously daily with 1 mg/kg tamoxifen (Sigma, St. Louis, MO, USA) dissolved in ethanol and suspended in sesame oil. The vehicle control group was injected daily with an equal volume of ethanol/sesame oil solvent. After 30 days of treatment, lymph-node-free right inguinal mammary glands were harvested, snap frozen and stored at -80°C for subsequent ECM isolation and biochemical analyses. To limit variations in ECM composition that may accompany oestrous cycling in the control group, phase of oestrous cycle was determined by daily cervical lavages and all control rats were sacrificed in the dioestrus 1 phase of the cycle, as previously described [29]. Cervical tissue was also collected for histological confirmation of oestrous cycle. The animal experiments were performed in duplicate. All animal procedures were performed in compliance with the AMC Cancer Research Institute and University of Colorado Denver Animal Care and Use Committees and National Institutes of Health (NIH) Policy on Humane Care and Use of Laboratory Animals.

### Primary fibroblast isolation

Left inguinal mammary glands (lymph nodes removed) were processed for fibroblast isolation immediately after sacrifice using a protocol provided by Kornelia Polyak which has been previously reported [30]. Briefly, the tissue was minced and incubated in collagenase. After incubation, the tissue was processed through a series of centrifugations to separate fibroblasts. Isolated cells were confirmed to be fibroblast-like when they tested negative for pan-cadherin and pan-cytokeratin and positive for vimentin by Western blot analyses (data not shown), and are referred herein as fibroblasts; however, endothelial cell specific markers were not evaluated. Fibroblasts, used at passage three, were maintained in Dulbecco's modified eagle medium (DMEM)/F12 media (Hyclone, Logan, UT, USA) supplemented with 25 mM Hepes (Sigma, St. Louis, MO, USA), 2 mM L-glutamine (Sigma, St. Louis, MO, USA), 50 μg/mL gentamicin (Sigma, St. Louis, MO, USA) and 20% FCS (Sigma, St. Louis, MO, USA).

### Fibroblast motility assay

Transwell 8 μm pore filters in a 24-well plate format (Becton Dickinson, Franklin Lakes, NJ, USA) were overlaid with 50 μL bovine gelatin, type B (Sigma, St Louis, MO, USA) at a concentration of 10 μg/mL and dried overnight. Five × 10^4 ^log-phase primary fibroblasts at passage three were suspended in incomplete media and plated into the upper chamber. The lower chamber contained 1% fetal bovine serum as chemoattractant. The number of motile cells, evaluated four hours after plating, was quantified as previously described [15]. The assay was performed in quadruplicate and data are expressed as mean ± standard error of the mean (SEM).

### Fibroblast-derived 3-dimensional matrices

Primary mammary fibroblasts were pooled according to group, and 2 × 10^5 ^cells were plated into six-well plates pre-coated with 50 μl of 2 μg/ml rat tail collagen (BD Biosciences, Bedford, MA, USA). Fibroblasts were cultured for 10 days in complete media, which was changed every two days. To isolate underlying ECM, fibroblasts were lysed in 20 mM ammonium hydroxide and 0.5% Triton X-100 followed by several washes in PBS and water, according to the published protocol by Cukierman [31]. The cell-free underlying ECM was scraped from plates and stored at -80°C for proteomic analysis.

### Histology

Whole mount mammary glands were fixed in methacarn, processed in a series of ethanol and xylene (Fisher, Fair Lawn, New Jersey, USA), stained with alum carmine (Fisher, Fair Lawn, New Jersey, USA), and bagged in methyl salicylate (Fisher, Fair Lawn, New Jersey, USA), as previously described [32]. Cervical tissue was also harvested, fixed in 10% neutral buffered formalin (NBF) for 18 hours, paraffin embedded, cut into 5 μm sections and stained with H&E. To control for morphological differences due to natural variation along the proximal/distal mammary gland axis, mammary tissue associated with the dissected lymph regions of gland number 4 were fixed in 10% NBF for 18 hours and processed for H&E staining as described above. The changes in mammary ductal and alveolar morphology in response to tamoxifen treatment were evaluated in these 5 μm H&E-stained sections as previously described [32].

For detection of fibrillar collagen, 5 μm sections of mammary tissue were stained with sirius red F3B according to published methods [33] and counterstained with Weigert's iron haematoxylin. Mammary gland collagen staining was classified into three grades. Grade 1 glands had fat pads composed primarily (more than 50%) of adipocytes and were free of dense interlobular collagen. Grade 2 glands had mixed fat pad morphology, with both adipocyte and fibrillar collagen-rich regions. Grade 3 glands had stroma dominated by fibrillar collagen. Collagen scoring was performed on coded slides by two independent investigators.

### Immunohistochemistry

Samples of 4 μm tissue were pretreated in 10 mM sodium citrate at 90°C for 20 minutes. Bromodeoxyuridine (BrdU; BD Immunocytometry Systems, San Jose, CA, USA, for rat tissue; Dako, Santa Barbara, CA, USA, for mouse tissue) and the macrophage marker CD68 (Serotec, Oxford, UK) were detected with a standard avidin biotin complex-peroxidase method with 3,3'-diaminobenzidine as the chromagen.

### Mammary ECM isolation

Mammary ECM isolation was performed based on a previously described protocol [5], using pooled inguinal mammary gland tissue from at least six rats per group. Briefly, frozen inguinal mammary glands, with lymph nodes removed, were pulverised, homogenised and extracted in a high salt/N-ethylmaleimide solution (3.4 M sodium chloride, 50 mM Tris-hydrochloric acid pH 7.4, 4 mM ethylenediaminetetraacetic acid (EDTA), 2 mM N-ethylmaleimide) containing protease inhibitor cocktail (100 μg/ml phenylmethylsulphonyl fluoride, 50 μg/ml each of aprotinin, leupeptin and pepstatin), at 4°C (chemicals purchased from Sigma, St Louis, MO, USA). Homogenates were enriched for ECM by two cycles of centrifugation (relative centrifugal force (RCF)_max _110,000 × g, 30 minutes, 4°C). The ECM-enriched pellets were resuspended in mid-salt/urea solution with proteinase inhibitor cocktail and extracted overnight at 4°C. Samples were pelleted at RCF_max _110,000 × g, and the ECM-enriched supernatants extensively dialysed (MWCO 12–14,000 kDa, Spectrum) against low salt buffer followed by dialysis against sera-free media (DMEM/F12 media (Sigma, St Louis, MO, USA) supplemented with 1 μg/ml gentamicin) at 4°C. ECM were stored on ice at 4°C and used within two weeks of isolation. As reported previously, ECM protein integrity is stable under these storage conditions [5]. For the *in vitro *studies, all experiments were performed with two distinct batches of ECM in at least duplicate.

### ECM proteomics

Aliquots of the mammary ECM isolates were subjected to in-solution tryptic digestion for label-free mass spectrometry-based analysis. Each sample was reduced and alkylated with 5 mM dithiothreitol and 20 mM iodoacetamide after the addition of Rapigest (Waters Technologies, Millford, MA, USA) to 0.1%. Trypsinisation was carried out overnight at 37°C and the reaction stopped by adding formic acid to a final concentration of 1%. Centrifugation was used to remove the hydrolytic detergent and sample zip tipped (Millipore, Temecula, CA, USA) to remove salts.

For electrospray ionisation mass spectrometry, each of the samples (2 μL) was injected onto a reverse-phase column using a chilled (9°C) autosampler (Eksigent; Dublin, CA, USA) connected to a high-performance liquid chromatography system run at 0.12 μL/minute before a passive split that resulted in about a 400 nL/minute post-split flow (Aligent; Santa Clara, CA, USA). A gradient of 12% to 30% acetonitrile (40 minutes) was applied over a two-hour run for peptide separation. The column effluent was coupled directly to a LTQ XL Linear Ion Trap mass spectrometer (Thermo/Finnigan; San Jose, CA, USA) with an in-house built nanospray ion source. Data acquisition was performed using the Xcalibur (version 2.0.6) software supplied with the instrument. The 60 minute liquid chromatography (LC) runs were monitored by sequentially recording the precursor scan (MS) followed by three collision-induced dissociation acquisitions (MS/MS). Singly charged ions were excluded from collision induced disassociation (CID) selection. Normalised collision energies were employed using helium as the collision gas. An in-house script was used to create de-isotoped centroided peak lists from the raw spectra (mgf format). These peak lists were searched against the SwissProt (V51.6) database using an in-house developmental Protein Prospector LC Batch-Tag Web (Version 4.25.2, UCSF, San Francisco, CA, USA) and an in-house Mascot server (Version 2.2, Matrix Science, Boston, MA, USA). For searches mass tolerances were ± 0.6 Da for MS peaks (+1 ^13^C option), and ± 0.6 Da for MS/MS fragment ions. Trypsin specificity was used allowing for one missed cleavage. The modifications of met oxidation, protein N-terminal acetylation, peptide N-terminal pyroglutamic acid formation were allowed for. Samples were searched against all rat and mouse entries in the protein database.

### Western blot analysis

Tissues from mammary glands were analysed by Western Blot as previously described [5]. The following antibodies were used: polyclonal rabbit anti-rat fibronectin (1:250 Life Technologies, Gaithersburg, MD, USA), polyclonal rabbit anti-laminin (1:500 Novus Biologicals, Littleton, CO, USA), mouse anti-laminin 5 (γ2 chain) monoclonal antibody (1:1000, Chemicon International, Temecula, CA, USA), collagen 1 (1:1000, Abcam, Cambridge, MA, USA) and protein A secondary antibody (1:10,000 Amersham, Piscataway, NJ, USA). Monoclonal mouse anti-glyceraldehyde 3-phosphate dehydrogenase (GAPDH; 1:500 Amersham, Piscataway, NJ, USA) followed by an anti-mouse secondary (1:5,000 Santa Cruz Biotechnology, Santa Cruz, CA, USA) was used for protein loading controls. Signal was obtained using ECL western detection kit (Amersham, Piscataway, NJ, USA). For fibronectin and collagen 1 Westerns, 3.3 μg of respective tissue samples were loaded per lane. For laminin (LN) westerns and zymogen assays, 9.5 μg of respective tissue samples were loaded per lane.

### Zymogen assay

Substrate gel analyses were performed as described [15]. Briefly, ECM samples loaded by equal protein were electrophoresed under non-reducing conditions on a 7.5% SDS-PAGE containing 3 mg/ml porcine gelatin (Sigma, St. Louis, MO, USA). Gels were incubated at 37°C for 72 hours in substrate buffer. Proteinase activity was visualised by Coomassie Blue 250 staining. Zymogen activity appears as a cleared band in a dark background. Semi-quantitative data was obtained by scanning densitometry of four independent lanes per condition with data analysed using BioRad Quantity One software (Bio-Rad, Hercules, CA, USA).

### Cell lines

J774 murine macrophage cells, a generous gift from Dr Douglas Graham, were cultured in DMEM/high glucose medium (Hyclone Laboratories, Logan, Utah, USA) supplemented with 10% heat-inactivated FCS as previously described [34]. MCF-12A cells are a non-tumourigenic human immortalised mammary epithelial cell line [35]. V12 Ras-transformed MCF-12A cells were previously described [36]. MCF-12A and MCF-12A-ras cells were grown in complete media consisting of Ham's F12/DMEM (Gibco, Carlsbad, CA, USA) containing 100 ng/ml cholera toxin (Gibco/BRL, Carlsbad, CA, USA), 0.5 μg/ml hydrocortisone (Sigma, St. Louis, MO, USA), 10 μg/ml insulin (Sigma, St. Louis, MO, USA), 20 ng/ml epidermal growth factor (EGF) (Sigma, St. Louis, MO, USA) and 5% horse serum (Gibco/BRL, Carlsbad, CA, USA). MDA-MB-231 cells (ATCC, Manassas, VA, USA), a human breast cancer cell line, were passaged into nude mice (mammary fat pad) and back out to plastic for at least four cycles as previously described [17]. The resulting variant cell line, which was enriched in the ability to grow in the fat pad was carried in MEM Alpha Media (Gibco, Carlsbad, CA, USA) completed with 2.2 g/L of sodium bicarbonate, 1% Hepes, 1% L-glutamine, 10% heat inactivated FCS, 1 μg/ml of insulin, 1% sodium pyruvate and 1% non-essential amino acids.

### Transwell filter assays

#### Motility assay

Log-phase murine macrophage J774 cells (1 × 10^5^) were suspended in 200 μL of DMEM/high-glucose medium (Hyclone Laboratories, Logan, Utah, USA) and plated on transwell 8 μm pore filters in a 24-well plate format (Becton Dickinson, Franklin Lakes, NJ, USA). The lower chamber contained 800 μL of 10 μg/mL control or tamoxifen mammary matrix with or without 10 μg/mL of rat tail collagen type1 (BD Biosciences, Bedford, MA, USA), 10 μg/mL of bovine skin gelatin, type B (Sigma, St. Louis, MO, USA) or 10 μg/mL of human plasma fibronectin (Collaborative Biochemical, Bedford, MA, USA). Eight hours post-plating, cells were fixed with 10% NBF, stained with 0.1% crystal violet and number of motile cells quantitated as previously described [15]. For tumour cell motility assays, log phase MDA-MB-231 cells (5 × 10^4 ^cells) were suspended in 10 μg/mL control or tamoxifen mammary gland ECM, and overlaid onto transwell 8 μm pore filters as described above. In the lower chamber, 1.0% horse serum was used as a chemoattractant and number of motile cells evaluated after 21 hours after plating. Motility assays were performed in quadruplicate and data are expressed as mean ± SEM.

#### Invasion assay

Transwell 8 μm pore filters in 24-well plate format (BD Biosciences, Bedford, MA, USA), were overlaid with 50 μL control or tamoxifen mammary ECM at a concentration of 200 μg/mL and dried overnight. Log-phase MCF12A-ras cells (5 × 10^4 ^cells), were suspended in incomplete media and plated into the upper chamber. The lower chamber contained 0.5% horse serum as chemoattractant. The number of invasive cells, evaluated 24 hours post plating, was quantified as previously described [15]. The assay was performed in quadruplicate and data are expressed as mean ± SEM.

#### Haptotaxis assay

Transwell 8 μm pore filters were underlaid (coated on the bottom of the filter) with 50 μL control or tamoxifen mammary ECM at a concentration of 75 μg/mL and dried overnight. Log-phase MDA-MB-231 cells (5 × 10^4 ^cells) were suspended in incomplete media and plated into the upper chamber. The lower chamber contained incomplete media; the ECM on the filter served as chemoattractant. The number of invasive cells, evaluated four hours post plating, was quantified as described above. For all transwell filter assays, differences between control and experimental conditions were determined using the two-tailed t-test.

### 3-dimensional culture model

Ras-transformed MCF-12A and MDA-MB-231 cells were cultured in 3D culture as previously described [5]. Briefly, log-phase cells were harvested and overlaid onto 2 mm thick matrix pads at cell concentrations of 4.5 × 10^4 ^(MCF-12A) or 1.5 × 10^4 ^(MDA-MB-231) or pre-coated with respective matrices with and without 20 μg/mL fibronectin (Collaborative Biochemical, Bedford, MA, USA) before overlaying onto the matrix pad, using a 96-well format (Sarstedt, Newton, NC, USA). The matrix substratum was composed of 50 μL of nulliparous or tamoxifen-treated rat mammary gland ECM (normalised for total protein concentration) mixed 1:1 with Matrigel (BD Biosciences, San Jose, CA, USA) to facilitate polymerisation of endogenous mammary ECM. For control conditions, the pad was composed of Matrigel, without endogenous mammary ECM and controlled for total protein concentration. For MCF-12A cells, 5% horse serum was added to the matrix pads. 3D culture assays were performed in triplicate and each experiment performed in duplicate with two different batches of endogenous mammary ECM.

### Image acquisition and quantitation

Five micron sections of 3D organoids capturing top, middle and bottom representative areas of the matrix pad were H&E stained and imaged at 20× using an Aperio Scan Scope T3 System, (Vista, CA, USA) at a resolution of 1 pixel/0.5 *u *and then down-sampled to a resolution of 1 pixel/2.4 *u*. Primary identification of cell clusters (organoids) was accomplished using NIH ImageJ (version 1.41o with Java 1.6.0_10.) that had been customised for colorimetric, size and circularity threshold gating steps using custom written plug-ins in the Java programming language. These image analysis algorithms were designed to identify and quantify immunohistochemical and morphological characteristics of 3D organoids. Image pre-processing procedures were applied to identify and remove from subsequent analyses images with scanning and staining errors. Images that passed these filters were analysed using a series of algorithms. The primary image algorithms relied on identifying histochemical features through colorimetric thresholds. Secondary pass morphometric quantifications focused on area and circularity measurements for feature identification. This automated algorithmic-targeted image analysis was utilised to eliminate intra-observer and inter-observer variability and results in a repeatable mathematically quantifiable set of data. The number of organoids analysed was 1456 in the Matrigel group, 1516 in the control mammary ECM group and 709 in the tamoxifen mammary ECM group. Mean cell cluster sizes were compared across treatment groups. Given that the data were not normally distributed, significance was evaluated using the Kruskal-Wallis, non-parametric test. Alternatively, 308 tamoxifen organoids and 241 tamoxifen plus fibronectin organoid images were captured under light microscopy and NIH ImageJ (version 1.41o with Java 1.6.0_10.) utilised to determine the average area per organoid, and differences determined using the Mann-Whitney U Test.

### Xenograft model

Forty eight-week old female homozygous Nu/Nu athymic nude mice (NCI, Frederick, MD, USA) were randomised by weight into two groups of 20. The animals were anaesthetised using isoflurane (Minrad, Inc., Bethlehem, PA, USA). Log-phase MDA-MB-231 cells were mixed with 270 μg/mL of control or tamoxifen rat mammary ECM at a concentration of 1.0 × 10^5 ^cells/μL matrix. Of the cell/matrix mix (2 × 10^6 ^total cells), 20 μL were back-loaded into a 3/10 cc insulin syringe with a 29 gauge 1/2 inch needle (BD Biosciences, San Jose, CA, USA) and injected into the fat pad of mammary gland number 4 [37]. For fibronectin experiments, MDA-MB-231 cells were mixed with incomplete α-MEM media with or without 20 μg/ml fibronectin (Collaborative Biochemical, Bedford, MA, USA) just before fat pad injection, with 10 mice per group. Tumour growth was measured using calipers twice weekly. Animals were sacrificed at six weeks post tumour cell injection. Tumours were excised, weighed and final tumour volume calculated (4/3πR^2^h). Statistical analyses were performed using the Kruskal-Wallis test and the Wilcoxon nonparametric analysis.

## Results

### Characterisation of mammary and cervical tissue in tamoxifen-treated rats

**Figure 1:**
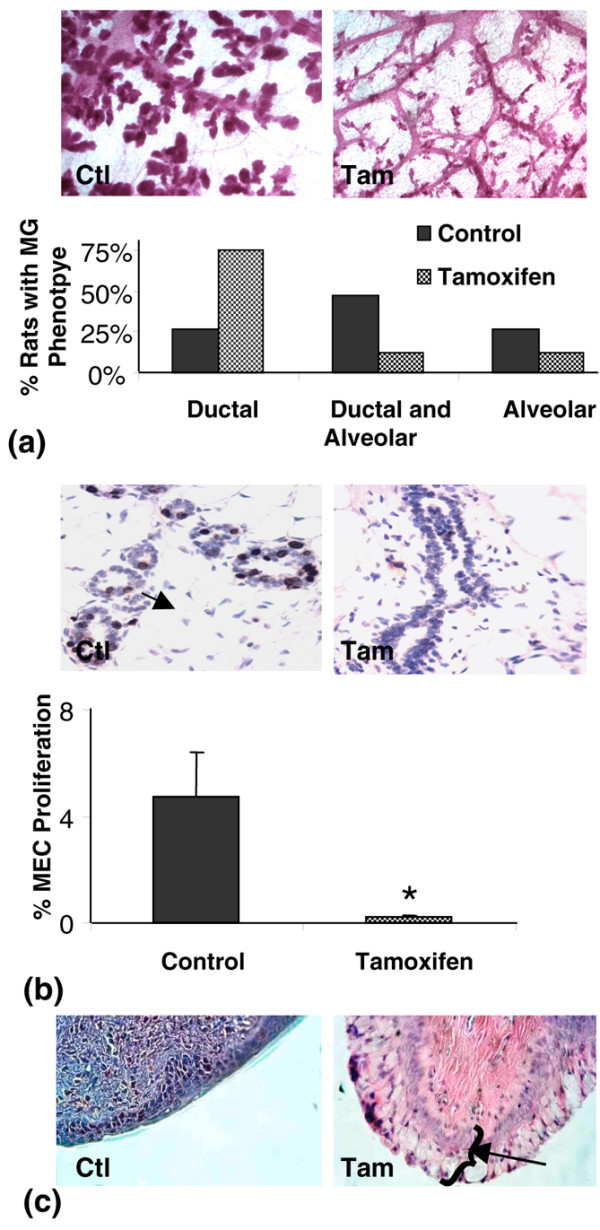
Effects of tamoxifen on proliferation of mammary and cervical epithelium. **(a) **upper panels: Whole mount mammary gland micrographs from control (Ctl) and tamoxifen-treated (Tam) rats showing loss of alveoli with tamoxifen treatment 40× magnification. **(a) **lower panel: Graph depicting percentage of rats in each group with dominant ductal, mixed or alveolar mammary gland morphology. **(b) **upper panels: Bromodeoxyuridine (BrdU) positive proliferating cells detected by immunohistochemistry. Arrow shows brown staining BrdU-positive cell. 400× magnification. **(b) **lower panel: Quantitation of proliferating mammary epithelial cells in control (n = 12) and tamoxifen (n = 12) treated rats, *p < 0.001. **(c) **Cervical histology of control and tamoxifen-treated rats. Arrow shows thick stratum germinativum layer in tamoxifen-treated rats consistent with hormone stimulation.

Tamoxifen is known to be an oestrogen antagonist in the mammary gland and acts to block proliferation of ER-positive epithelial cells. As expected, in comparison to control rats, the mammary epithelium in the tamoxifen-treated rats had a significant reduction in alveolar development (Figure 1a, upper panels). Morphometric analysis demonstrated that only 25% of vehicle-treated rats had mammary glands lacking well-developed alveoli, whereas 75% of mammary glands from tamoxifen-treated rats had mammary glands lacking alveoli (Figure 1a, graph). Consistent with the loss of mammary alveoli in the tamoxifen-treated rats, mammary epithelial cell proliferation, as measured by BrdU incorporation, was significantly reduced (Figure 1b, upper panels; BrdU quantification is shown in Figure 1b, lower panel). In the uterus, tamoxifen is an oestrogen agonist and one of tamoxifen's known side effects in women is an increase in uterine tumours. As previously reported by others, we found that tamoxifen-treated rats did not cycle through oestrous and the uterine and cervical tissues from these rats displayed proliferative phenotypes (Figure 1c), indicating that, as in women, tamoxifen acts as an oestrogen agonist in the rat uterus [38].

### Characterisation of mammary stroma composition

**Figure 2:**
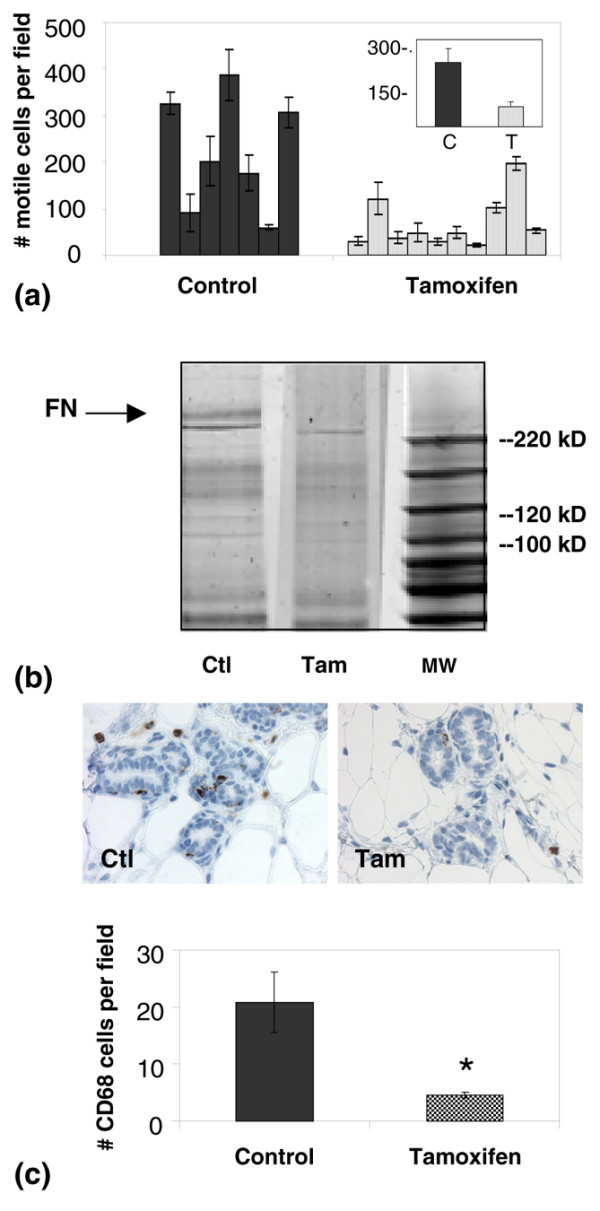
Influence of tamoxifen treatment on mammary stromal fibroblasts and macrophages. **(a) **Motility of primary mammary fibroblasts isolated from individual control rats (Ctl; n = 7), tamoxifen-treated rats (Tam; n = 10), and pooled populations (insert). **(b) **One-dimensional gel of extracellular matrix (ECM) proteins laid down in culture by primary mammary fibroblasts isolated from control and tamoxifen-treated rats. Arrow shows about a prominent 250 kD band identified as containing fibronectin (FN) by mass spectrometry, which is present in control ECM and greatly reduced in tamoxifen ECM. Lane 3 depicts molecular weight (MW) marker. **(c) **upper panel: 5 μm representative sections of mammary glands stained for CD68, a macrophage lysosomal-associated protein. 400× magnification. **(c) **lower panel: Quantitation of CD68 positive mammary cells in control (n = 6) and tamoxifen-treated (n = 6) rats demonstrates significantly fewer macrophages with tamoxifen treatment (*p = 0.013).

To begin to address the question of whether tamoxifen alters mammary stroma, primary fibroblasts were isolated from mammary glands of control and tamoxifen-treated rats. Fibroblast cells were characterised as positive by immunohistochemistry staining for vimentin and smooth muscle actin and by lack of pan-cadherin staining (data not shown). Fibroblast motility was assessed as a functional marker of cell activity. Although inter-animal variation in fibroblast motility was high, overall mammary fibroblasts isolated from the tamoxifen-treated animals displayed a significant three-fold decrease in transwell filter motility compared with control mammary fibroblasts (Figure 2a).

Fibroblasts are the major producers of mammary interlobular and periductal ECM and the motility data suggested that ECM production might be similarly altered by tamoxifen treatment. To address this question, the ECM secreted by primary fibroblasts in culture and incorporated into a 3D insoluble matrix was harvested and run on a 1D-gel. A prominent band of about 250 kD was decreased in the tamoxifen group (Figure 2b, arrow). This band was excised and found to contain fibronectin by mass spectrometry. These data show that *in vitro*, mammary fibroblasts isolated from tamoxifen-treated rats are less motile and incorporate less fibronectin into the insoluble matrix than fibroblasts isolated from vehicle control rats, data consistent with tamoxifen treatment causing fibroblast quiescence.

**Figure 3:**
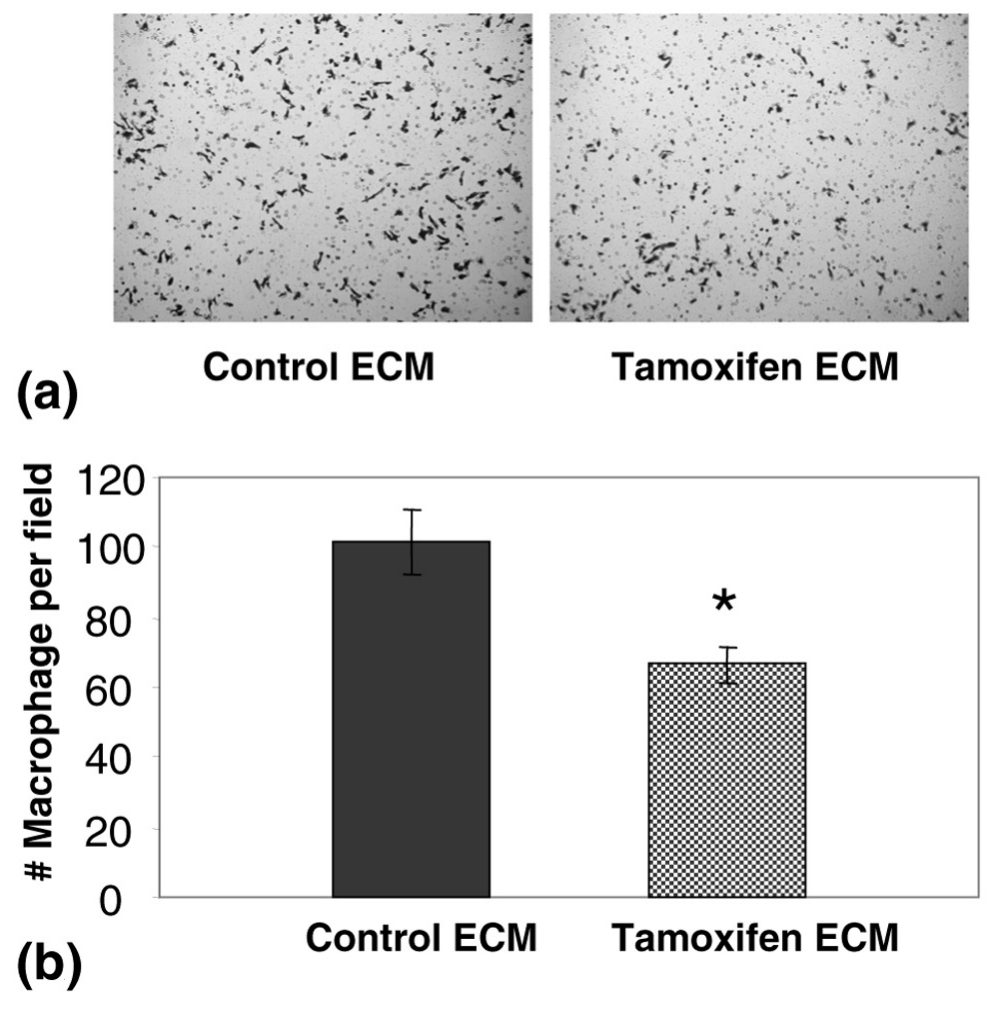
Tamoxifen extracellular matrix (ECM) recruits fewer macrophages than control ECM in a transwell filter assay. **(a) **Photomicrographs of transwell filters showing that J774 macrophage cells are less chemotactic towards tamoxifen ECM compared with control ECM. 100× magnification. **(b) **Quantitation of J774 cell motility demonstrates significantly reduced chemotaxis towards tamoxifen ECM. *p = 0.003.

Tissue macrophages are a stromal cell type whose presence correlates directly with breast cancer progression in women and in rodent models [39]. To determine whether tamoxifen treatment influenced the macrophage content of the mammary gland, immunohistochemistry analysis of the macrophage lysosomal marker CD68 was performed (Figure 2c, upper panels). Quantification of CD68 staining demonstrated that the CD68 signal was significantly decreased in mammary glands of tamoxifen-treated rats (Figure 2c, graph). Given that ECM proteins can function as attractants for macrophages, we next determined whether isolated ECM preparations from control and tamoxifen-treated rats differentially attract macrophages in a transwell filter assay. Using the isolated mammary matrices as chemoattractants, the motility of J774 cells, a mouse macrophage cell line, was suppressed by tamoxifen ECM in comparison with control ECM (Figure 3). These observations suggest that changes in mammary ECM composition with tamoxifen treatment could provide a plausible mechanism by which macrophage number is reduced in the mammary glands of tamoxifen-treated rats.

### Tissue matrix proteomics

To identify prominent ECM proteins that compose the rat mammary gland stroma and which might be differentially expressed with tamoxifen treatment, ECM proteins were extracted from mammary glands of control and tamoxifen-treated rats and identified by tandem mass spectrometry. Prominent ECM proteins identified included fibronectin, collagen chains α-1(I), α-2(XIV) and α-2(I), laminin chains α4, β1, β2 and γ1, nidogen 1 and fibrillin 1. In addition, numerous matrix proteoglycans were identified in the mammary ECM, including decorin, perlecan, lumican, biglycan, mimecan and periostin (Table 1).

**Table 1:** Mammary ECM proteins identified by mass spectrometry

**Swiss-Prot accession #**	**Protein**
[Swiss-Prot:Q80X19]	Collagen alpha-1(XIV)
[Swiss-Prot:P02454]	Collagen alpha-1(I)
[Swiss-Prot:P02466]	Collagen alpha-2(I)
[Swiss-Prot:Q61554]	Fibrillin 1
[Swiss-Prot:Q9WVH8]	Fibulin-5
[Swiss-Prot:P04937]	Fibronectin
[Swiss-Prot:P97927]	Laminin subunit alpha-4
[Swiss-Prot:P02469]	Laminin subunit beta-1
[Swiss-Prot:P15800]	Laminin subunit beta-2
[Swiss-Prot:P15800]	Laminin subunit beta-2
[Swiss-Prot:P02468]	Laminin subunit gamma-1
[Swiss-Prot:P47853]	Biglycan
[Swiss-Prot:Q01129]	Decorin
[Swiss-Prot:Q9QZZ6]	Dermatopontin
[Swiss-Prot:P51886]	Lumican
[Swiss-Prot:Q62000]	Mimecan
[Swiss-Prot:Q05793]	Perlecan
[Swiss-Prot:P09650]	Mast cell protease 1
[Swiss-Prot:O88766]	Neutrophil collagenase
[Swiss-Prot:P10493]	Nidogen-1
[Swiss-Prot:Q62009]	Periostin
[Swiss-Prot:P29457]	Serpin H1 precursor

**Figure 4:**
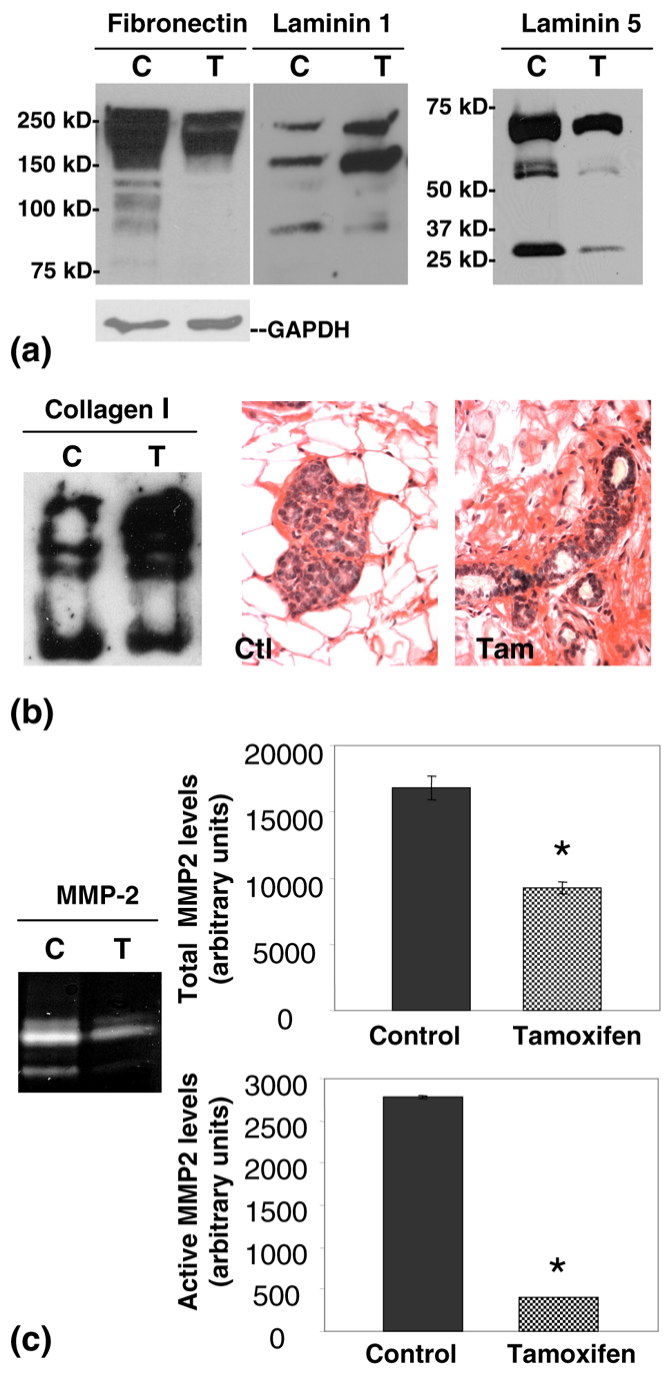
Mammary extracellular matrix (ECM) proteolysis is reduced with tamoxifen treatment. **(a) **Western blot analyses of fibronectin, laminin 1 and laminin 5 in mammary tissue from control and tamoxifen-treated rats, with glyceraldehyde 3-phosphate dehydrogenase (GAPDH) as loading control. **(b) **Fibrillar collagen detected by Western blot (left panel) and in 5 μm Picro-sirius red stained mammary gland sections. **(c) **Matrix metalloproteinase (MMP) 2 activity detected by zymography, with total MMP-2 levels and active MMP-2 levels quantified by scanning densitometry (*p = 0.005 and *p < 0.0001, respectively).

To determine whether the relative abundance of the identified mammary ECM proteins differed with tamoxifen treatment, several of the ECM proteins identified by proteomics were evaluated by Western blot. Mammary tissue from tamoxifen-treated rats had decreased levels of fibronectin (Figure 4a), data consistent with the *in vitro *fibroblast data described in Figure 2b. In addition, tamoxifen treatment resulted in a modest increase in the basement membrane protein laminin 1, and a decrease in total laminin 5 levels (Figure 4a). Western blot analysis revealed that tamoxifen treatment increased levels of the intra-lobular and inter-lobular ECM protein collagen 1 (Figure 4b, left panel). Tissue collagen deposition, as measured by Picro-sirius red staining, confirmed elevated levels of fibrillar collagen in the mammary stroma of tamoxifen-treated rats (Figure 4b, right panels and Table 2). Further, Western blot analyses demonstrated a generalised decrease in mammary ECM proteolysis with tamoxifen treatment, as evidenced by an increase in the ratio of high to low molecular weight species of fibronectin, laminin 1, laminin 5 and collagen 1 (Figures 4a, b).

**Table 2:** Fibrillar collagen content of mammary glands

**Grade of collagen deposition^a^**	**Control group**	**Tamoxifen group**
Grade I	5 (23%)	0 (0%)

Grade II	12 (54%)	9 (38%)

Grade III	5 (23%)	15 (62%)

^**a **^Grade I glands are at least 50% adipocyte-rich stroma and devoid of dense fibrillar collagen deposition. Grade II glands have moderate levels of dense fibrillar collagen or areas with mixed collagen densities and Grade III glands have at least 50% of glands filled with dense fibrillar collagen deposition.

Activity of MMP-2, an MMP found in high levels in virgin rat mammary glands and implicated in mammary ECM proteolysis, was evaluated in mammary tissue of control and tamoxifen-treated rats. Total MMP-2 and active MMP-2 were found to be decreased in mammary tissue of tamoxifen-treated rats (Figure 4c, left panel). Quantitation by densitometry showed about a 45% decrease in total MMP-2 (Figure 4c, upper right panel) and more than a 85% reduction in the cleaved active form of MMP-2 (Figure 4c, lower right panel). These data show that the decrease in ECM proteolysis in mammary glands of tamoxifen-treated rats correlated with concurrent decreases in MMP-2 levels and activity.

### *In vitro *functional evaluations of ECM

**Figure 5:**
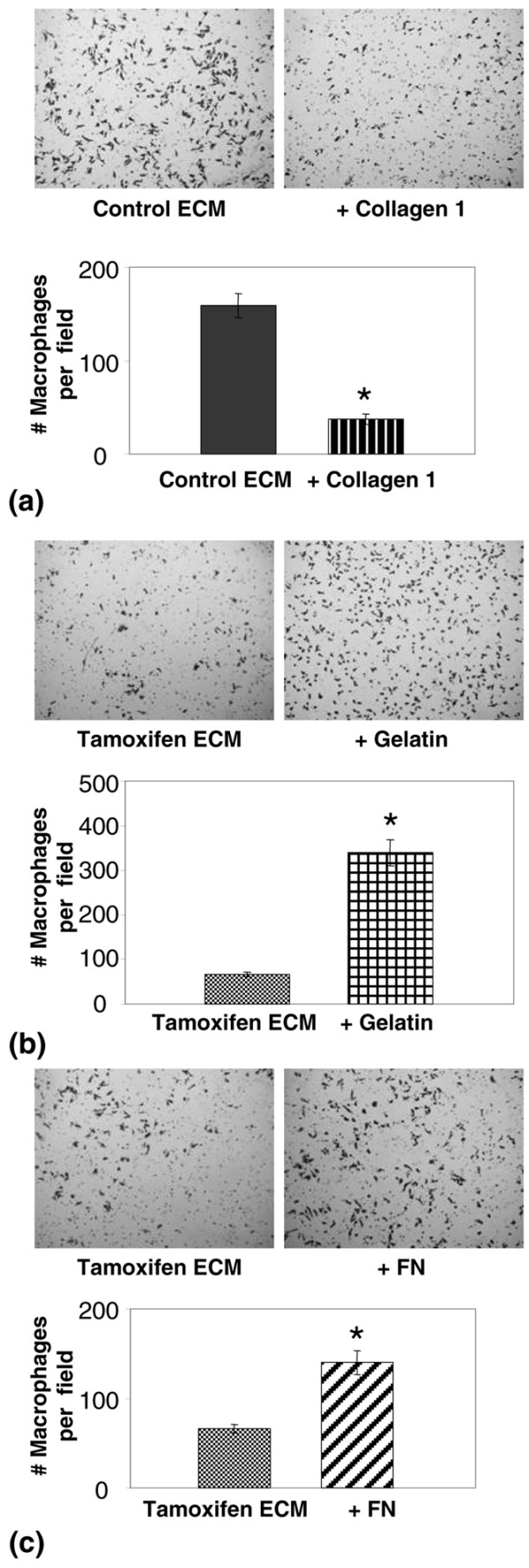
Macrophage chemotaxis is influenced by the denatured state of collagen 1 and fibronectin (FN) levels. **(a) **upper panel: Photomicrograph of transwell filter showing inhibition of J774 cell motility when 10 μg/ml fibrillar collagen 1 is added to control extracellular matrix (ECM) and used as chemoattractant. 100× magnification. **(a) **lower panel: Quantitation of data demonstrates significantly reduced chemotaxis towards collagen 1. *p < 0.0001. **(b) **upper panel: Photomicrograph showing that 10 μg/ml denatured collagen (gelatin) added to tamoxifen ECM as chemoattractant increases J774 macrophage cell motility. 100× magnification. **(b) **lower panel: Quantitation of data demonstrates significantly increased chemotaxis towards denatured collagen, *p < 0.0001. **(c) **upper panel: Photomicrograph showing that 20 μg/ml FN added to tamoxifen ECM increases J774 cell motility. 100 × magnification. **(c) **lower panel: quantitation of data demonstrates significantly increased chemotaxis towards FN. *p = 0.0004.

As confirmed by Western blot analyses, prominent differences between control and tamoxifen mammary ECM composition were the presence of high levels of intact fibrillar collagen in mammary tissue of tamoxifen-treated rats and the presence of partially proteolysed collagen and elevated levels of fibronectin in control mammary glands. The question of whether these ECM components influence macrophage motility was evaluated next. To reconstitute the high levels of collagen 1 observed in tamoxifen ECM, 10 μg/ml fibrillar collagen 1 was added to control ECM. The addition of fibrillar collagen was found to suppress J774 macrophage cell motility compared with control ECM alone (Figure 5a). Conversely, the addition of 10 μg/ml denatured collagen to tamoxifen mammary ECM was found to stimulate macrophage motility (Figure 5b). Given that fibronectin levels were higher in control ECM than tamoxifen ECM, the effect of 10 μg/ml fibronectin on macrophage motility was also investigated. We found that the addition of fibronectin to tamoxifen ECM had a modest but statistically significant stimulatory influence on J774 cell motility when compared with tamoxifen ECM alone (Figure 5c). Cumulatively, these data suggest that the high level of intact collagen and reduced level of fibronectin in the mammary tissue of tamoxifen-treated rats contributes to the reduced number of macrophages observed in mammary glands of tamoxifen-treated rats.

**Figure 6:**
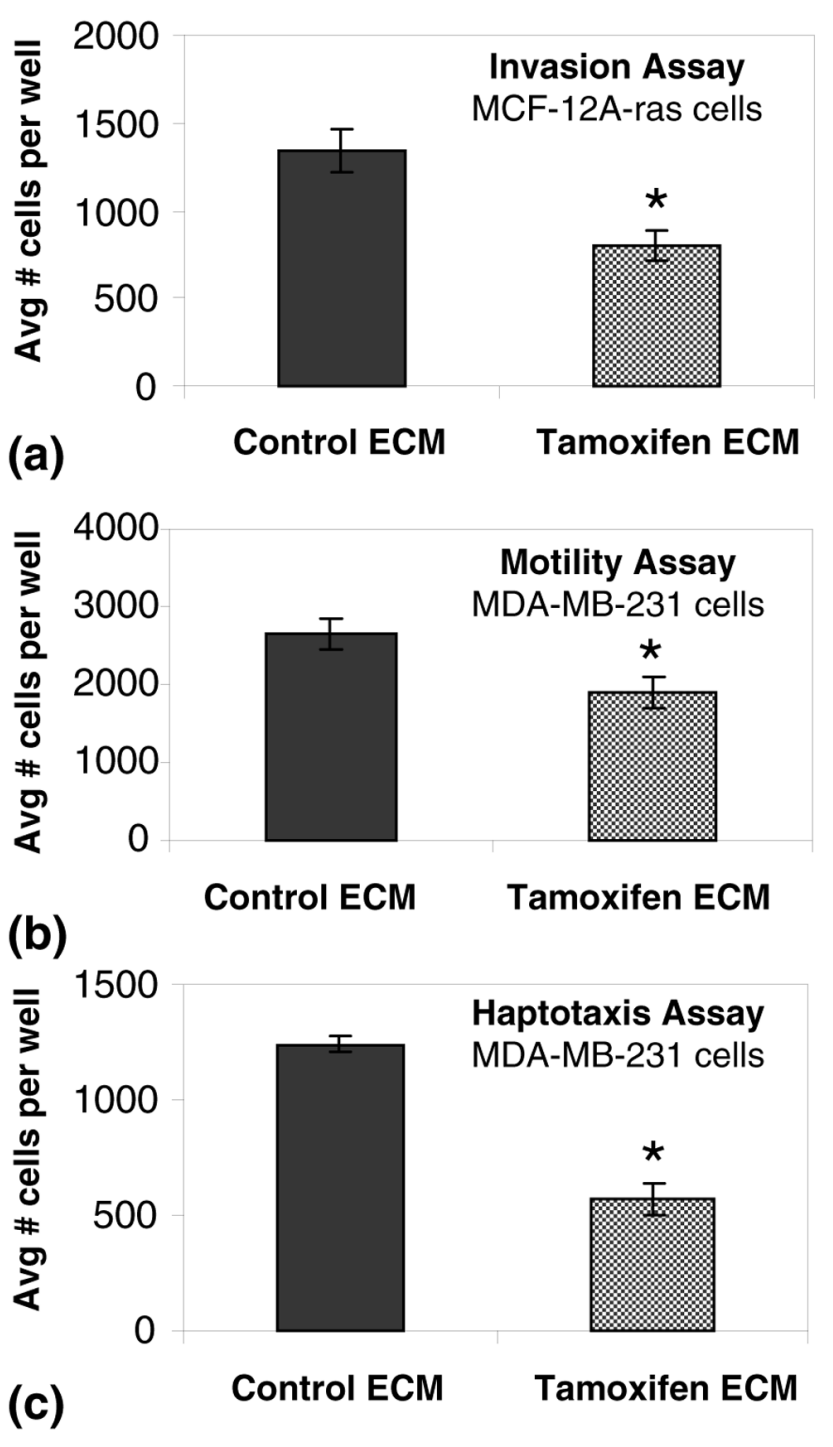
Mammary extracellular matrix (ECM) from tamoxifen-treated rats suppresses metastatic attributes of transformed mammary epithelial cells in comparison with ECM from control rats. **(a) **MCF-12A-Ras cell invasion through 8 μm transwell filter pores occluded with 200 μg/ml mammary ECM was suppressed in response to tamoxifen in comparison with control ECM. *p < 0.024. **(b) **Motility of MDA-MB-231 cells was inhibited in response to filters coated with 10 μg/ml tamoxifen ECM compared with control ECM. *p < 0.005. **(c) **Using mammary matrices as chemoattractant, MDA-MB-231 cells were less motile towards tamoxifen ECM. *p < 8.3E-5.

To address whether mammary ECM isolated from tamoxifen-treated rats inhibits metastatic-attributes of epithelial tumour cells *in vitro*, the effects of control and tamoxifen ECM on mammary tumour cell invasion, motility and haptotaxis were evaluated. In a transwell filter assay, the invasiveness of Ras transformed mammary epithelial MCF-12A cells was reduced two-fold on tamoxifen ECM compared with control ECM (Figure 6a). Differences in motility were not observed (data not shown). These functional analyses were extended to the highly metastatic human breast cancer MDA-MB-231 cells. MDA-MB-231 cells had reduced motility on the tamoxifen ECM (Figure 6b), but no change in invasion (data not shown). The haptotactic properties of the isolated mammary ECM were evaluated using mammary ECM as the chemoattractant. MDA-MB-231 cells displayed a three-fold reduction in haptotactic properties towards tamoxifen ECM (Figure 6c). Cumulatively, these data suggest a partial suppression of the metastatic phenotype by tamoxifen ECM.

**Figure 7:**
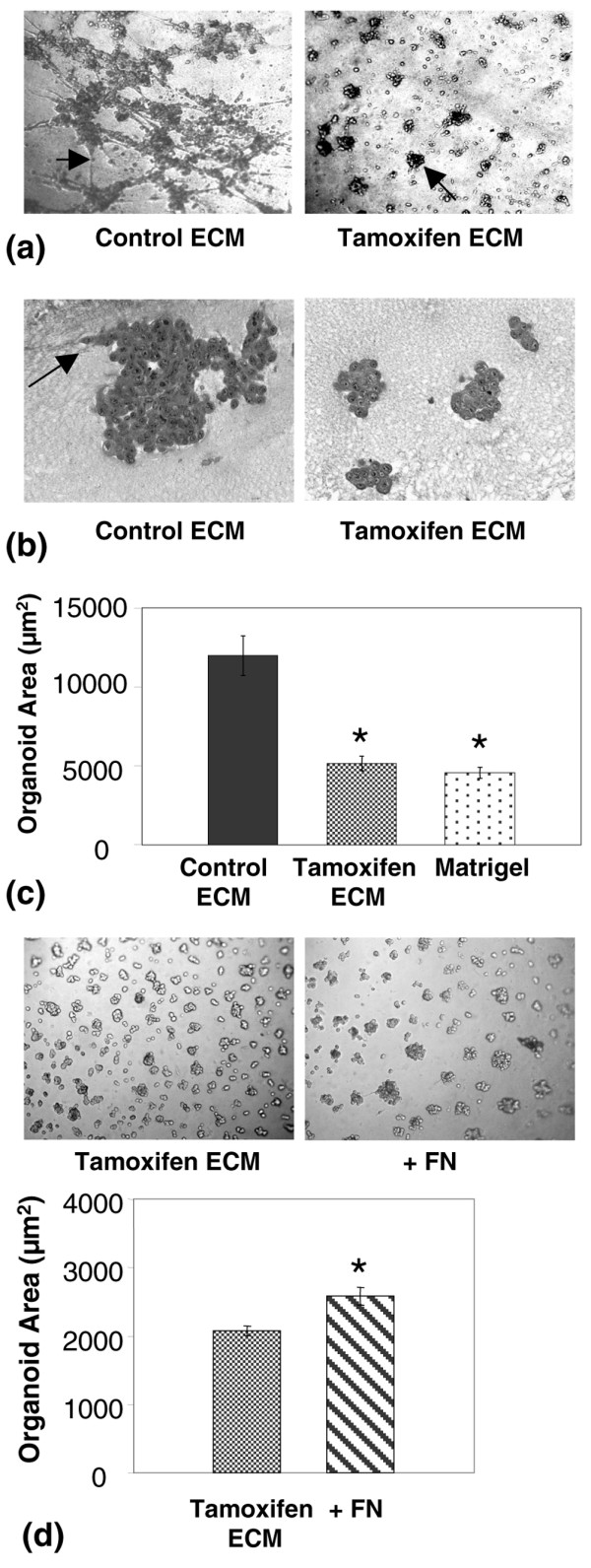
Mammary extracellular matrix (ECM) isolated from tamoxifen-treated rats inhibits tumour cell invasion in 3-dimensional (3D) culture. **(a) **Whole-mount 3D organoid images of MCF12A-Ras cells plated onto control and tamoxifen derived mammary ECM. Left panel arrow depicts commonly occurring tumour cell filapodia invading control ECM. Right panel shows small compacted 3D organoids that formed on mammary ECM isolated from tamoxifen treated rats. **(b) **5 μm H&E-stained sections of organoids on control and tamoxifen ECM 200× magnification. Arrow depicts locally invasive tumour cells present in control ECM cultures. **(c) **Quantitation of organoid size that developed on control mammary ECM, tamoxifen ECM and Matrigel. Tamoxifen and Matrigel organoid size do not differ, but are different from control ECM organoid size. *p < 0.0001. **(d) **upper panel: The addition of 20 μg/ml fibronectin (FN) to tamoxifen ECM increased organoid size by about 20%. **(d) **lower panel: Quantitation of organoid size. *p < 0.002.

To further characterise the effects of these ECM matrices on tumour cell behaviour, 3D cell culture assays were performed. When plated onto control ECM, MCF-12A-Ras cells displayed an aggressive phenotype characterised by disorganised structuring and invasive filapodia (Figure 7a, left panel). In contrast, when plated onto tamoxifen ECM, these same cells formed compacted smooth-surfaced multicellular clusters lacking invasive filapodia (Figure 7a, right panel). H&E stained 4 μm sections of 3D organoids confirmed the observation that tamoxifen ECM induced small spheroid shaped organoids (Figure 7b). Quantitative morphometric analyses of organoid size demonstrated that the average size of organoids plated onto tamoxifen ECM was 2.6-fold smaller than organoids on control ECM (Figure 7c). Even very aggressive MDA-MB-231 cells had a subtly altered 3D phenotype on the tamoxifen ECM. These cells were more clustered and cuboidal in appearance than when plated on the control ECM, indicating induction of an epithelial-like morphology (data not shown). The 3D culture data corroborate the transwell filter assay data and show that the tamoxifen ECM fails to support tumour cell motility and invasion.

To evaluate the contributions of individual ECM proteins to tumour organoid formation, reconstitution experiments were performed with fibronectin and collagen 1. The addition of high molecular weight fibronectin to tamoxifen matrix was found to consistently increase organoid size in 3D culture, but the increase in size was modest (Figure 7d). Further, an increase in local invasion was observed with fibronectin (data not shown). These data suggest that an increase in fibronectin alone does not wholly account for the aggressive phenotype displayed by MCF-12A-Ras cells plated on control ECM. Of interest, the addition of fibrillar collagen to control matrix disrupted 3D organoid formation, thus the contribution of collagen to the suppressive microenvironment could not be evaluated in this model (data not shown).

### *In vivo *functional evaluation of ECM

**Figure 8:**
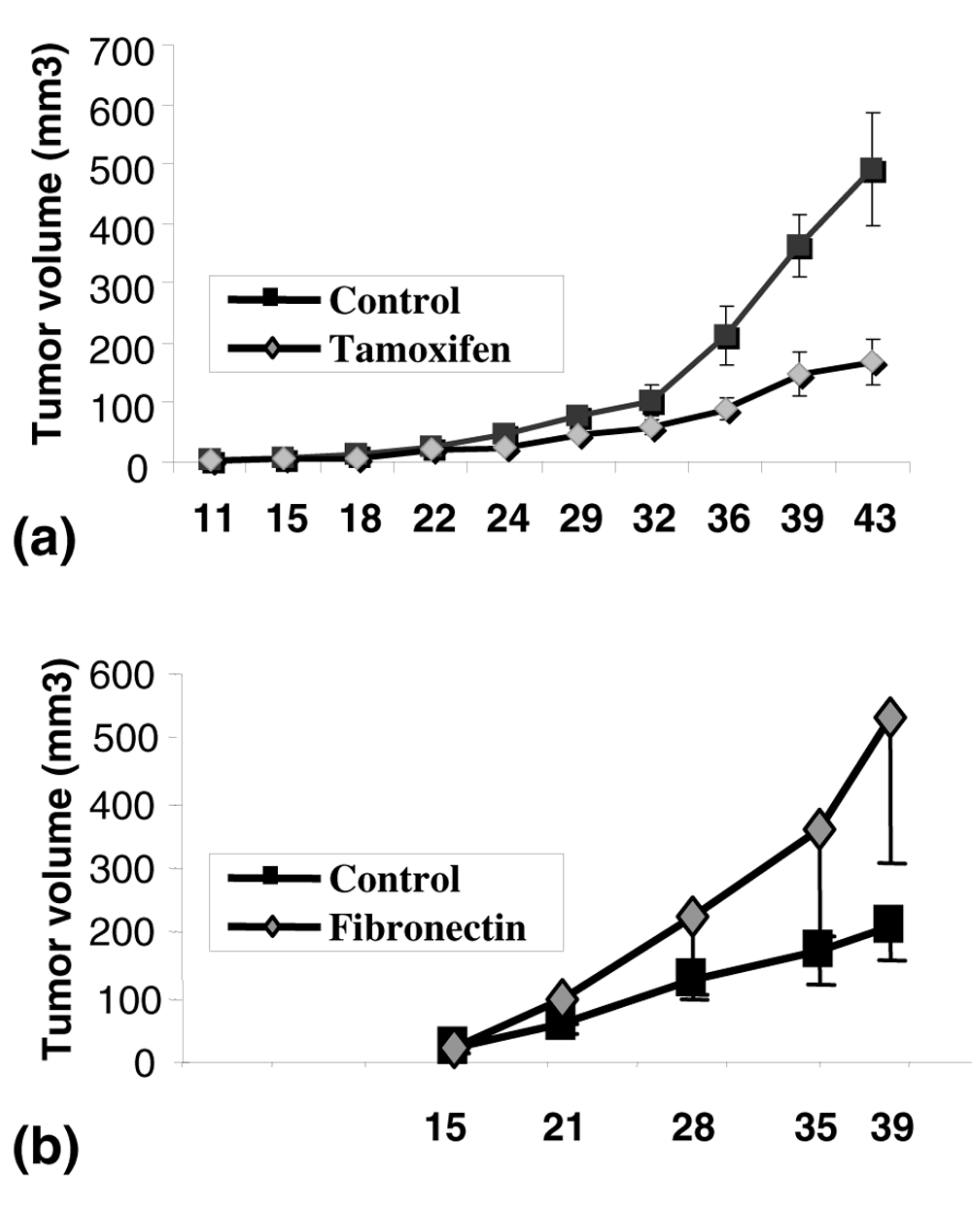
Mammary extracellular matrix (ECM) isolated from tamoxifen-treated rats inhibits tumours in an orthotopic xenograft model. **(a) **MDA-MB-231 tumour cells mixed with tamoxifen ECM formed smaller tumours than when mixed with control ECM. n = 20 per group. **(b) **MDA-MB-231 tumour cells mixed with 20 μg/ml fibronectin had a trend towards larger tumour size, consistent with fibronectin contributing to larger tumour size observed with control ECM. n = 10 per group.

Based on the demonstration that tamoxifen ECM reduced the aggressive characteristics of two transformed breast cancer cell lines in multiple cell culture models, we investigated whether tamoxifen ECM could decrease tumour promotion *in vivo*. For this study, MDA-MB-231 cells were mixed with mammary ECM from control and tamoxifen-treated rats and injected into the inguinal fat pads of female athymic mice. By seven weeks after tumour cell injection, tumour incidence was the same between groups; however, mice injected with tumour cells plus tamoxifen ECM had tumours that were three times smaller than those injected with tumour cells mixed with control ECM (Figure 8a). To determine whether the elevated levels of fibronectin in control mammary ECM influence tumour size in this xenograft model, as suggested by the 3D organoid data, MDA-MB-231 cells were treated with 20 μg/ml fibronectin before mammary fat pad injection (Figure 8b). Tumour size was increased by fibronectin across all time points, however, because of a large variation in tumour size within a group, these data did not reach statistical significance.

## Discussion

In this paper we characterised the tissue microenvironment associated with quiescent mammary epithelium. Mammary quiescence was induced with the anti-oestrogen tamoxifen, an agent known to suppress proliferation of normal mammary epithelium and ER-positive tumour cells [40]. In our study, in addition to the expected anti-proliferative effects of tamoxifen on mammary epithelium, tamoxifen treatment was found to induce pleiotrophic changes in the mammary microenvironment. These changes included inhibition of fibroblast motility and fibronectin incorporation into the substratum, a decrease in macrophage number, a reduction in MMP-2 activity and markedly less proteolysis of the ECM proteins fibronectin, laminin 1, laminin 5 and collagen 1. This response of the mammary stroma to tamoxifen treatment provides further evidence for the hypothesis that dynamic and reciprocal interactions occur between epithelium and stroma, and that these interactions dictate epithelial cell function [4,41]. Further, this study demonstrates the plasticity of the adult mammary stroma, which has significant implications for stromal-targeted therapeutics.

ECM proteins represent a dominant component of the stromal microenvironment and ECM composition, organisation, cross linking and turnover are attributes determined primarily by stromal cells. Thus, ECM composition can be considered as a read-out of changes in stromal cell function. As such, we evaluated whether tamoxifen treatment altered functional attributes of mammary ECM. Matrix proteins isolated from tamoxifen-treated rats were found to suppress macrophage and breast tumour cell motility, tumour cell invasion and haptotaxis in transwell filter assays, to significantly reduce the size of tumour cell organoids in 3D culture, and to inhibit tumour progression in a xenograft model of breast cancer. Mammary ECM isolated from control rats did not have these attributes. These data indicate that tamoxifen modifies the mammary microenvironment in a manner consistent with tumour suppression, which may shed light on tumour dormancy, the latent phase that occurs between treatment and disease progression. Relapse from dormancy can occur after prolonged periods of disease-free survival, and is a particular problem in breast cancer [20,21].

Using tamoxifen-induced mammary quiescence as an *in vivo *model for tumour dormancy, we identified several candidate stromal mediators of tumour quiescence. We observed a decrease in total fibronectin in mammary tissue with tamoxifen treatment and reduced fibronectin secretion by fibroblasts isolated from mammary glands of tamoxifen-treated rats. In rodent models, fibronectin mRNA expression is upregulated during periods of high proliferation such as puberty and pregnancy, and suppressed during gland involution following weaning [5,6,42]. Further, the addition of fibronectin to Matrigel in a 3D culture model stimulated mammary epithelial cell proliferation and increased acinar size [43]. These observations implicate fibronectin as a key ECM mediator of mammary epithelial cell proliferation *in vivo*. Further, under adverse conditions such as hypoxia, fibronectin can act as a pro-survival protein due to upregulation of α5β1 integrin. For example, oncogenic activation of the receptor tyrosine kinase ERBB2 contributes to tumour cell resistance to hypoxia by inducing the α5β1 integrin fibronectin receptor, which increases cell adhesion and survival [44]. In a pancreatic tumour model, fibronectin interaction with α5β3 caused transactivation of the insulin-like growth factor-1 receptor and increased tumour cell survival [45]. Additional roles for fibronectin in promoting tumour cells are suggested by data demonstrating that proteolytic processing of fibronectin induces breast tumour cell motility, invasion and activation of MMP-9 [15,16].

Fibronectin has also been implicated directly in the induction and regulation of dormancy. In cell culture studies, downregulation of β1 integrin induced invasive fibroblast-like breast tumour cells to revert to polarised cells capable of contributing to acinar-like epithelium *in vitro *[24]. In a head and neck carcinoma model, α5β1 integrin engagement with fibronectin matrix promoted tumour cell proliferation, whereas disruption of this ECM interaction suppressed extracellular signal-regulated kinase activation and induced a dormant-like state [46,47]. In another 3D model of dormancy, using several breast cancer cell lines that exhibit dormancy in lungs of xenografted mice, the transition from quiescence to proliferation was found to be dependent on fibronectin production, signaling through β1 integrin, cytoskeletal reorganisation and the formation of actin stress fibers [48]. Thus, the reconstitution experiments reported here, where exogenous fibronectin partially reverted the protective effect of tamoxifen ECM on tumour cells *in vitro *and in a xenograft model of breast cancer, implicate reduced levels of fibronectin as being a signal from the microenvironment that contributes to tumour cell suppression. Our data indicate that this important growth promoting ECM signal is negatively regulated by tamoxifen.

An additional prominent difference between tamoxifen-treated and control rat mammary stroma was the reduction in matrix proteolysis in the tamoxifen group. Proteolytic cleavage of ECM proteins, which can unmask cryptic sites and release fragments with bioactivity distinct from the parent molecule, have important roles in immune modulation and cancer progression [49-51]. For example, *in vitro *collagen 1, fibronectin, laminin and entactin derived peptides exhibit chemotactic activity for inflammatory cells [49,52]. These previously published studies are consistent with our data showing macrophage number was reduced in tamoxifen-treated mammary glands, *in vitro *macrophage motility suppressed by tamoxifen matrix and macrophage motility stimulated by the addition of exogenous fibronectin and denatured collagen; ECM proteins prominent in control mammary matrix. The observation that tamoxifen treatment reduces mammary macrophage number is intriguing because macrophages have been demonstrated to be required for the rapid proliferative phase that occurs with pubertal gland expansion [53]. In addition, macrophages are implicated in numerous events associated with cancer progression, including enhanced breast tumour cell motility, increased ECM remodeling, and blood vessel development [53-55]. In women, breast cancers with a macrophage infiltrate have a poorer prognosis, supporting the hypothesis that macrophages are promoters of breast cancer [56]. Our data extends these observations to suggest that inhibition of ECM remodeling and macrophage recruitment are components of the quiescent/dormant mammary microenvironment.

Tamoxifen treatment was also associated with an apparent inhibition of LN5 γ2 chain cleavage because the level of about a 25 kD proteolytic fragment of γ2 was reduced with tamoxifen treatment. It has previously been demonstrated that membrane type 1 (MT1)-MMP cleaves the rat γ2 chain to release about a 30 kD fragment containing EGF-like repeats [57]. This LN5 fragment binds to EGF receptors and stimulates breast tumour cell scattering and migration. A similar proteolytic cleavage site and bioactive γ2 fragment has been identified in human LN5 [51]. The apparent ability of tamoxifen to reduce the amount of this pro-tumourigenic LN5 fragment is additional support for suppression of ECM turnover being important for gland quiescence.

Collagen type 1 chains α-1(I) and α-2(I), components of fibrillar collagen, were identified in the isolated ECM by proteomic analysis. Further, fibrillar collagen, detected in tissue sections by Sirius red F3B staining, was observed at higher levels in tamoxifen-treated glands than in control glands. This observation is somewhat perplexing because high levels of fibrillar collagen are a component of reactive stroma around tumours and, *in vivo*, breast cancer tumour cells have been observed to migrate along collagen fibrils during metastasis [58,59]. As with fibronectin and laminin, mammary collagen 1 cleavage was decreased with tamoxifen treatment (Figure 4b). Thus, slower matrix turnover may account for the high levels of fibrillar collagen observed with tamoxifen, but this remains to be determined. Collagen turnover has been correlated with a tumour permissive stroma, whereas inhibition of collagen turnover has been shown to delay mammary tumour progression and metastasis [60,61]. Using human melanoma cells, cell-cycle arrest was induced by fibrillar collagen [62]. The question of whether mammary epithelial cell contact with fibrillar collagen similarly restricts proliferation has not been evaluated.

Our proteomic analysis identified several proteoglycans present in mammary ECM including decorin, lumican, perlecan, biglycan, mimecan and periostin. These proteoglycans have roles in collagen assembly, fibrosis, wound healing and cancer [63]. The roles of these ECM proteins in normal and transformed mammary tissue are largely unknown, and these proteins remain interesting candidates for further study.

The pleiotrophic response of the mammary stroma to tamoxifen treatment suggests that tamoxifen influences the expression of a master regulator of stromal function. Research by other groups indicates that tumour growth factor (TGF) β, which acts on fibroblasts, immune and endothelial cells, in addition to epithelial cells, is a candidate regulator. In tamoxifen-sensitive breast tumour cells, growth inhibition has been shown to be mediated by activation of TGFβ [64]. Further, the ability of tumour cells to upregulate TGFβ and its receptor TβRII predict drug sensitivity [28]. The question of whether TGFβ mediates the response to tamoxifen in normal glandular epithelium, as reported here, is unknown. Another question unresolved by our study is whether the response of the stroma to tamoxifen is direct or indirect. ER-positive mammary fibroblasts have been described and, in our study, a small percentage of the mammary fibroblasts were found by immunohistochemistry to be ERα or ERβ positive (data not shown). Direct evidence for breast tumour fibroblasts modulating tamoxifen sensitivity in tumour cells has recently been reported [65]. In that study, using a 3D co-culture model, MCF-7 cells mixed with Erα-positive/progesterone receptor (PR)-positive fibroblasts were growth inhibited by tamoxifen, whereas MCF-7 cells co-cultured with Erα-negative/PR-negative fibroblasts exhibited decreased tamoxifen sensitivity [65]. That study is highly supportive of tumour fibroblasts being direct targets of tamoxifen.

## Conclusions

In summary, in the normal mammary gland, a homeostatic balance appears to occur between ECM synthesis and turnover resulting in the regulated release of bioactive ECM fragments. One potential function of these ECM fragments is the recruitment of macrophages for immune surveillance. Based on our *in vitro *and *in vivo *studies, we find that the ECM turnover and macrophage recruitment associated with the normal mammary microenvironment is supportive of tumour cell progression. Tamoxifen treatment was found to shift this balance toward suppression of fibronectin synthesis, reduced ECM turnover, accumulation of fibrillar collagen and reduced macrophage infiltrate, resulting in a microenvironment that was suppressive to breast tumour cells. Cumulatively, these data implicate suppression of ECM turnover as being critical for a quiescent mammary microenvironment and possibly tumour dormancy. Also, these data suggest the compelling possibility that the microenvironment may be part of the response to a variety of physiological and drug interventions that are chemoprotective, and that by characterising stromal changes induced by these agents, important insight into tumour cell dormancy can be obtained.

## Abbreviations

3D: three-dimensional; BrdU: bromodeoxyuridine; DMEM: Dulbecco's modified eagle medium; ECM: extracellular matrix; EDTA: ethylenediaminetetraacetic acid; EGF: epidermal growth factor; EGFR: epidermal growth factor receptor; ER: oestrogen receptor; FCS: fetal calf serum; GAPDH: glyceraldehyde 3-phosphate dehydrogenase; H&E: haematoxylin and eosin; LN: laminin; MEC: mammary gland epithelial cells; MG: mammary gland; MMP: matrix metalloproteinase; MT1-MMP: membrane type 1 matrix metalloproteinase; MW: molecular weight; NBF: neutral buffered formalin; NIH: National Institutes of Health; PBS: phosphate buffered saline; PR: progesterone receptor; RCF: relative centrifugal force; SEM: standard error of the mean; TGFβ: tumour growth factor beta.

## Competing interests

The authors declare that they have no competing interests.

## Authors' contributions

RH participated in study design, animal husbandry, performed most cell culture experiments and mammary ECM isolation. RH was also involved in statistical analyses, drafting of the manuscript and production of figures. OM performed all macrophage experiments, 3D organoid quantitation, ECM reconstitution experiments and production of figures. SM was involved in study design, animal husbandry, primary fibroblast isolation and motility assays, and mammary ECM isolation. KCH was responsible for mass spectrometry studies and mining of mass spectrometry data. KJH was responsible for animal husbandry, Western blot and zymography analyses. TL performed the fibronectin xenograft study. SL developed the automated morphometric quantitation system required for the 3D assays. SW developed the software required for the automated quantisation of 3D organoids and performed subsequent analyses and statistical tests. PS was responsible for study conception, design, data interpretation and manuscript preparation.
